# Randomized study on the effectiveness of nomegestrol acetate plus 17β-estradiol oral contraceptive versus dienogest oral pill in women with suspected endometriosis‑associated chronic pelvic pain

**DOI:** 10.1186/s12905-022-01737-7

**Published:** 2022-05-10

**Authors:** Salvatore Caruso, Antonio Cianci, Marco Iraci Sareri, Marco Panella, Giuseppe Caruso, Stefano Cianci

**Affiliations:** 1 Department of General Surgery and Medical Surgical Specialties, Gynecological Clinic, University of Catania, Via Santa Sofia 78, 95123 Catania, Italy; 2 Research Group for Sexology, University of Catania, Catania, Italy; 3 Department BIOMETEC, University of Catania, Catania, Italy; 4 Department of Obstetrics and Gynecology, University of Messina, Messina, Italy

**Keywords:** Hormonal contraceptives, Dienogest, Endometriosis-associated chronic pelvic pain, Nomegestrol acetate, Quality of life, Sexual function, 17β-estradiol

## Abstract

**Background:**

To evaluate the effects of a combined oral contraceptive containing 1.5 mg 17b-estradiol (E2) and 2.5 mg nomegestrol acetate (NOMAC) or 2 mg/daily dienogest (DNG) oral progestin on endometriosis-associated chronic pelvic pain (CPP) and on the quality of life (QoL) and sexual function, by a randomized study design.

**Methods:**

The E2/NOMAC group and DNG group included 99 and 98 women, respectively. The levels of CPP were measured by the visual analogic scale (VAS). The QoL scores were investigated by the Short Form-36 questionnaire (SF-36). Finally, sexual function was studied using the Female Sexual Function Index (FSFI), while sexual distress was studied by the Female Sexual Distress Scale (FSDS). The study had 3, 6 and 12-month follow-ups.

**Results:**

The intra-group analysis showed an improvement of the VAS score from baseline to the 12-month follow-up in the women of both groups (*p* < 0.001). The inter-group comparison showed a similar improvement of CPP (*p* = 0.06). Women on DNG had better SF-36 somatic (*p* < 0.01) and FSFI scores (*p* < 0.006) than women on E2/NOMAC at the 6- and 12-month follow-ups.

**Conclusions:**

The results support the efficacy of both hormonal treatments, even if DNG was more effective than E2/NOMAC in a limited intergroup comparison.

## Introduction

Chronic pelvic pain (CPP) due to endometriosis is a disabling symptom that affects about 10% of women of childbearing age [1]. Rather than adopting extended or continuous treatments to control their CPP, women use on-demand non-steroidal anti-inflammatory drugs (NSAIDs), obtaining transient and poor symptom resolution [2]. They begin to take hormonal therapies, usually combined hormonal contraceptives (CHC) or progestins, when they are diagnosed with endometriosis by their doctors [3, 4].

In fact, six national and two international guidelines agree that oral estrogen-progestins or progestins are the first-line medical option in cases of women with endometriosis‑associated CPP [5].

Several studies conducted on the efficacy of hormonal treatments in reducing endometriosis‑associated CPP have shown that it may depend on the type, dosage, route of administration and regimen for either the estrogen-progestins or the progestins alone. Moreover, most studies have investigated the efficacy of single-treatment, either estrogen-progestin or progestin, and there are very few comparative treatments [6]. Furthermore, women with different types of endometriosis have often been enrolled in a single study and treated with single hormone therapy, and, finally, for short-term follow-ups [7].

Among the most recent CHCs, a pill having 1.5 mg 17β-estradiol (E2) and 2.5 mg nomegestrol acetate (NOMAC) is used, with 24 days of active pill and a 4-day placebo pill regimen. The progestin activity of NOMAC is able to cover the 4-day hormone-free interval; this is because of its long half-life of 45–50 h [8].

Moreover, progestins are an alternative option for inhibiting estrogen-induced lesion proliferation and reducing endometriosis-associated CPP. Of the progestogens, dienogest (DNG) 2 mg per day is the only one approved for the clinical treatment of endometriosis [9]. The effects of DNG on endometriotic lesions are manifold; in fact, it has not only antiproliferative but also antiangiogenic and immunological activities [9]. A peculiarity of the aforementioned hormonal treatments is that they are effective as long as they are used. In fact, when the woman stops their intake, the endometriosis-associated symptoms could reappear.

The aim of this study was to evaluate the effects of E2/NOMAC and DNG on endometriosis-associated CPP (the primary endpoint) and on the quality of life (QoL) and sexual function (the secondary endpoints) of women, by a randomized study design.

## Methods

Based on the ESHRE guidelines [10], 347 women aged 18–39 years old (mean age 27.3 ± 7.8), affected by CPP, dysmenorrhea and dyspareunia were recruited to participate in this study. The time from onset of CPP ranged from 2 to 10 years. Each woman had been using on-demand non-steroidal anti-inflammatory drugs (NSAIDs) from 15 months to 8 years.

Before enrollment, the inclusion and exclusion criteria for CHC or only-progestin usage were assessed medically. Furthermore, the exclusion criteria included women who had been on GnRh or hormonal treatments within the previous 6 or 3 months, respectively; or affected by infertility; or a woman or her partner with sexual dysfunction; or not having any sexual activity. All included women underwent physical and gynecological examinations, and finally transvaginal sonography (TVS). Consequently, 114 (32.8%) women with clinical signs and TVS diagnosed with recto-vaginal endometriosis [44 (12.7%)], ovarian endometrioma [49 (14.1%)], or adenomyosis [21 (6%)] were excluded. They were included in a medical/surgical arm not considered in this study. Finally, 233 (67.2%) women received counseling on the benefits of E2/NOMAC intake in an extended 24/4 regimen and of daily progestogen DNG 2 mg. However, after counseling 36 (15.5%) women decided not to participate in the study, wanting to use NSAID on-demand therapy, while 197 (76.7%) women accepted to participate in the study. According to a computer-generated list to randomize participants 1:1, each woman was allocated to the E2/NOMAC group or DNG group.

Ethics Committee Catania 1, University Hospital Polyclinic, Catania, Italy, approved the study protocol. It conformed to the ethical guidelines of the 2013 Helsinki Declaration. Informed written consent was obtained from each woman before entering the study. None of them received any payments. The time of enrollment was from October 2017 to November 2019.

### Instruments

The Visual Analogic Scale (VAS) was used to define endometriosis-associated pain [11], such as CPP, dysmenorrhea and dyspareunia. The Short Form-36 (SF-36) questionnaire was used to assess QoL [12]. To assess sexual behavior, the self-administered Female Sexual Function Index (FSFI), validated in the Italian gynecological population, was used [13]. An FSFI cut-off of ≤ 26.55 is usually accepted for the diagnosis of sexual dysfunction in women within a wide age range. Moreover, for the diagnosis of sexual dysfunction, an essential element is the requirement that the condition causes significant personal distress for the woman. Therefore, the Female Sexual Distress Scale (FSDS) was used [14], having a cut-off of ≥ 15.

The study included three follow-ups at 3, 6 and 12 months. All questionnaires were administered to both groups at the baseline evaluation and at each follow-up.

### Statistical analysis

Assuming a standard deviation of 4 and a mean difference of 3 with a p ≤ 0.05 for the primary outcome measure (VAS), the sample size calculation indicated that 84 subjects would be the minimum number for each study arm required to have 95% statistically significant power.

Considering a dropout rate of 25–30% [15], 196 women were considered the number of subjects to be invited to participate in the study.

Intention-to-treat analyses were performed for all efficacy variables and included all women who had undergone the baseline evaluation and had at least one efficacy assessment after the baseline visit. The “last observation carried forward” method was used to select data such that missing data were replaced by values from the last available assessment during treatment before the respective assessment. The $\chi^{2}$ and ANOVA tests were used to compare the demographic and clinical data between the two groups, respectively. The difference was estimated with the 95% confidence interval (CI). To quantitatively measure the strength of statistical significance between two groups having similar standard deviations and size, the effect size was calculated by Cohen's d. Paired Student’s t-test was used to compare the values obtained at baseline with those of the follow-ups from the SF-36 domains and VAS. For comparisons of the values obtained from the FSFI items between baseline and the follow-ups, the nonparametric Wilcoxon rank-sum test with z values was used. Scores are presented as mean ± SD. The correlation analyses with Pearson’s r coefficient were performed to examine the relationships between the VAS and FSFI scores. The result was statistically significant when *p* < 0.05. The effect size was defined as small, medium, large, or very large when Cohen’s d was 0.2, 0.5, 0.8, or 1.3, respectively. Statistical analysis was carried out using the Primer of Biostatistics statistical computer package (Glantz SA, New York, USA: McGraw-Hill, Inc. 1997).

## Results

Women were randomized to the E2/NOMAC group (n.99) or the DNG group (n.98). Table 1 shows the demographic characteristics of both groups at baseline.

**Table 1:** Demographic characteristics

	E2/NOMAC group n = 99	DNG group n = 98	P
Age range (years)	18 to 38	18 to 39	1
Mean age	26.4 ± 6.8	27.4 ± 8.3	0.3
BMI kg/m^2^	21.8 ± 4.7	22.1 ± 3.5	0.6
Age at menarche	12.6 ± 2.4	12.8 ± 3.1	0.6
Menstrual cycle length (days)	26 to 32	26 to 33	1
Duration of menses (days)	4.5 ± 2.2	4.1 ± 1.8	0.1
Chronic Pelvic Pain	99 (100%)	98 (100%)	1
Time from onset ranged (years)	2 to 10	2 to 10	1
Dysmenorrhea	73 (73.7%)	74 (75.5%)	0.9
Dyspareunia	62 (62.6%)	63 (64.3%)	1
Parity			
Nulliparous	74 (74.7%)	75 (76.5%)	0.9
One or more children	25 (25.3%)	23 (23.5%)	0.9
Cigarette smoking			
Non-smoker	84 (84.8%)	82 (83.7%)	0.9
Current smoker	15 (15.2%)	16 (16.3%)	0.7
Daily cigarettes	15.3 ± 3.2	14.7 ± 4.2	0.2
Systolic blood pressure (mmHg)	117.5 ± 9.5	119.2 ± 5.7	0.13
Diastolic blood pressure (mmHg)	71.3 ± 7.8	69.5 ± 7.5	0.1
Heart rate (× min)	72.5 ± 8.6	70.6 ± 5.5	0.06

At the 3-month follow-up, 10 (10.1%) women of the E2/NOMAC group discontinued due to irregular bleeding; the dropout rate was 12.1% (twelve women) and 11.2% (eleven women) at the 6- and 12-month follow-up, respectively. Therefore, 66 (66.6%) women completed the study. On the other hand, 6 (6.2%) and 4 (4.1%) women on DNG discontinued treatment due to irregular bleeding at the 3- and the 6-month follow-ups, respectively. At the 12-month follow-up, the dropout rate was 14.7% (thirteen women). Therefore, 75 (76.5%) women completed the study. Intention-to-treat analyses were performed as reported in the statistical analysis. In addition, in their daily diary the women on E2/NOMAC recorded mild adverse events at the 3-month follow-up, which did not provoke discontinuation, namely spotting (16 = 17.9%), nausea (12 = 135%), or breast tenderness (13 = 14.6%). Moreover, some women on DNG reported mild adverse events at the 3-month follow-up, which did not provoke discontinuation, namely spotting (17 = 18.4%), nausea (11 = 11.9%), or breast tenderness (15 = 16.3%). Finally, 17 (18.1%) and 20 (30.3%) women on E2/NOMAC, and 19 (20.6%) and 49 (65.3%) women on DNG reported amenorrhea at the 3- and the 12-month follow-ups, respectively.

**Fig. 1:**
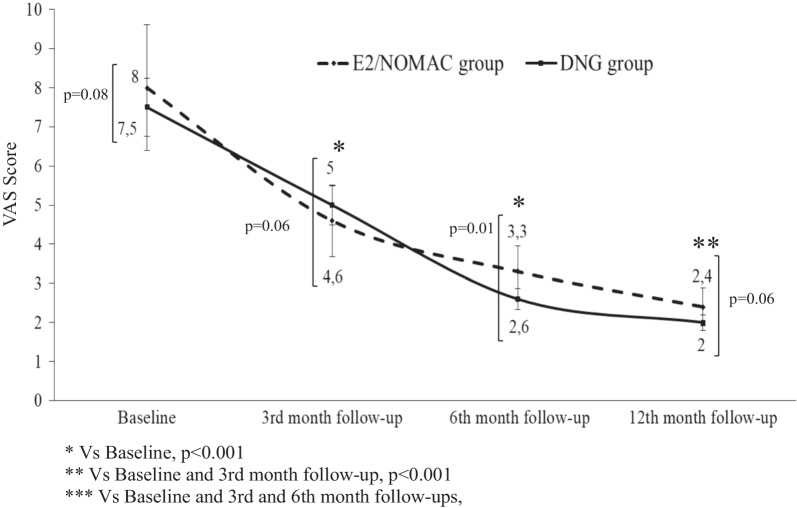
Visual Analog Scale (VAS) score of women affected by endometriosis-associated chronic pelvic pain at 3, 6 and 12 months of 24/4 regimen 17β-estradiol (1.5 mg) and Nomegestrol Acetate (2.5 mg) (E2/NOMAC) combined oral contraceptive, or of Dienogest (DNG) 2 mg daily

The intra-group analysis showed an improvement of the VAS score from baseline to the 12-month follow-up in women of both the E2/NOMAC and DNG groups (*p* < 0.001). The inter-group comparison showed no differences between baseline (*p* = 0.08; Cohen’s d = 0.5), and the 3- (*p* = 0.06; Cohen’s d = 0.8) and 12-month (*p* = 0.06; Cohen’s d = 0.7) follow-up values. On the contrary, at the 6-month follow-up women on DNG had a better improvement than women on E2/NOMAC (*p* = 0.01; Cohen’s d = 0.7) (Fig. 1).

**Fig. 2:**
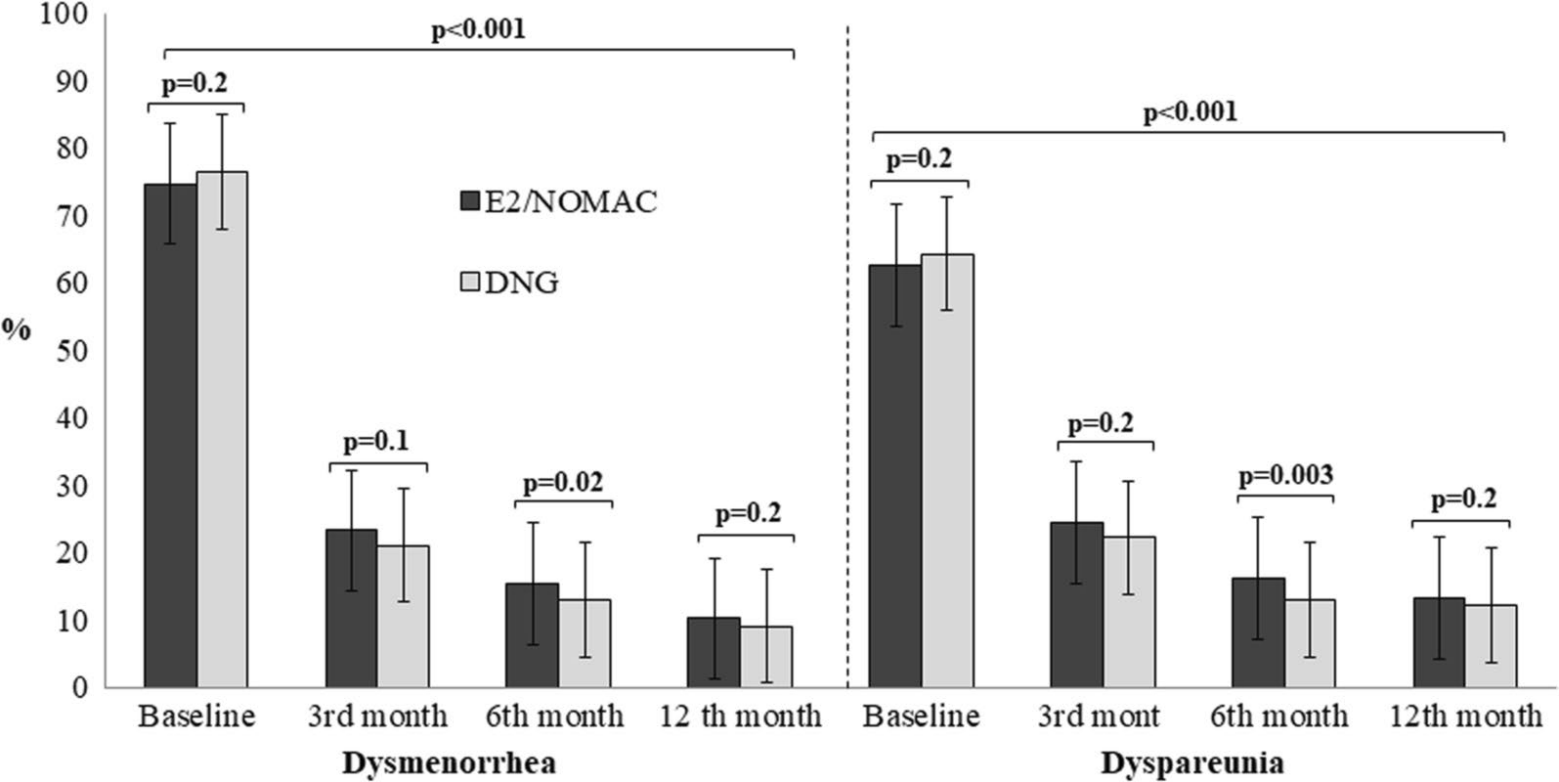
Dysmenorrhea and dyspareunia intragroup and intergroup comparison of women with endometriosis-associated pain symptoms at baseline and at 3, 6 and 12 months of 24/4 regimen 17β-estradiol (1.5 mg) and Nomegestrol Acetate (2.5 mg) (E2/NOMAC) combined oral contraceptive, or of Dienogest (DNG) 2 mg daily

Figure 2 shows dysmenorrhea and dyspareunia intragroup and intergroup comparison. Women of both groups had a significant reduction of dysmenorrhea and dyspareunia at each follow-up compared to baseline values (*p* < 0.001).

Table 2 shows the intergroup analysis at each follow-up for CPP, dysmenorrhea and dyspareunia. No difference was observed by intergroup analysis for dysmenorrhea at baseline (*p* = 0.2; Cohen’s d = 0.6), and at the 3- month (*p* = 0.1; Cohen’s d = 0.8) and 12-month follow-ups (*p* = 0.2; Cohen’s d = 0.7). Similarly, no differences were observed for dyspareunia at baseline (*p* = 0.2; Cohen’s d = 0.5) and at the 3- month (*p* = 0.2; Cohen’s d = 0.7) and 12-month follow-ups (*p* = 0.2; Cohen’s d = 0.5). At the 6-month follow-up, the DNG group had a better improvement than the E2/NOMAC group for both dysmenorrhea (*p* = 0.02; Cohen’s d = 1.8) and dyspareunia (*p* = 0.003; Cohen’s d = 2.5).

**Table 2:** Endometriosis-associated pain symptoms intergroup comparison at baseline and at 3, 6 and 12 months of women on 24/4 regimen 17β-estradiol (1.5 mg) and Nomegestrol Acetate (2.5 mg) (E2/NOMAC) combined oral contraceptive, or on Dienogest (DNG) 2 mg daily

Pain symptoms	Baseline		3rd month		6th month		12th month	
	95% CI	P*	95% CI	P*	95% CI	P*	95% CI	P*
Chronic Pelvic Pain	− 0.06 to 1.06	0.08	− 0.82 to 0.02	0.06	0.13 to 1.26	0.01	− 0.02 to 0.82	0.06
Dysmenorrhea	− 4.61 to 1.01	0.2	− 0.48 to 2.88	0.1	0.32 to 4.47	0.02	− 0.6 to 2.8	0.2
Dyspareunia	− 4.51 to 1.11	0.12	− 1.35 to 5.95	0.2	1.12 to 5.27	0.003	− 0.96 to 2.96	0.2

*t = two-sided *t* test; CI = Confidence interval

**Fig. 3:**
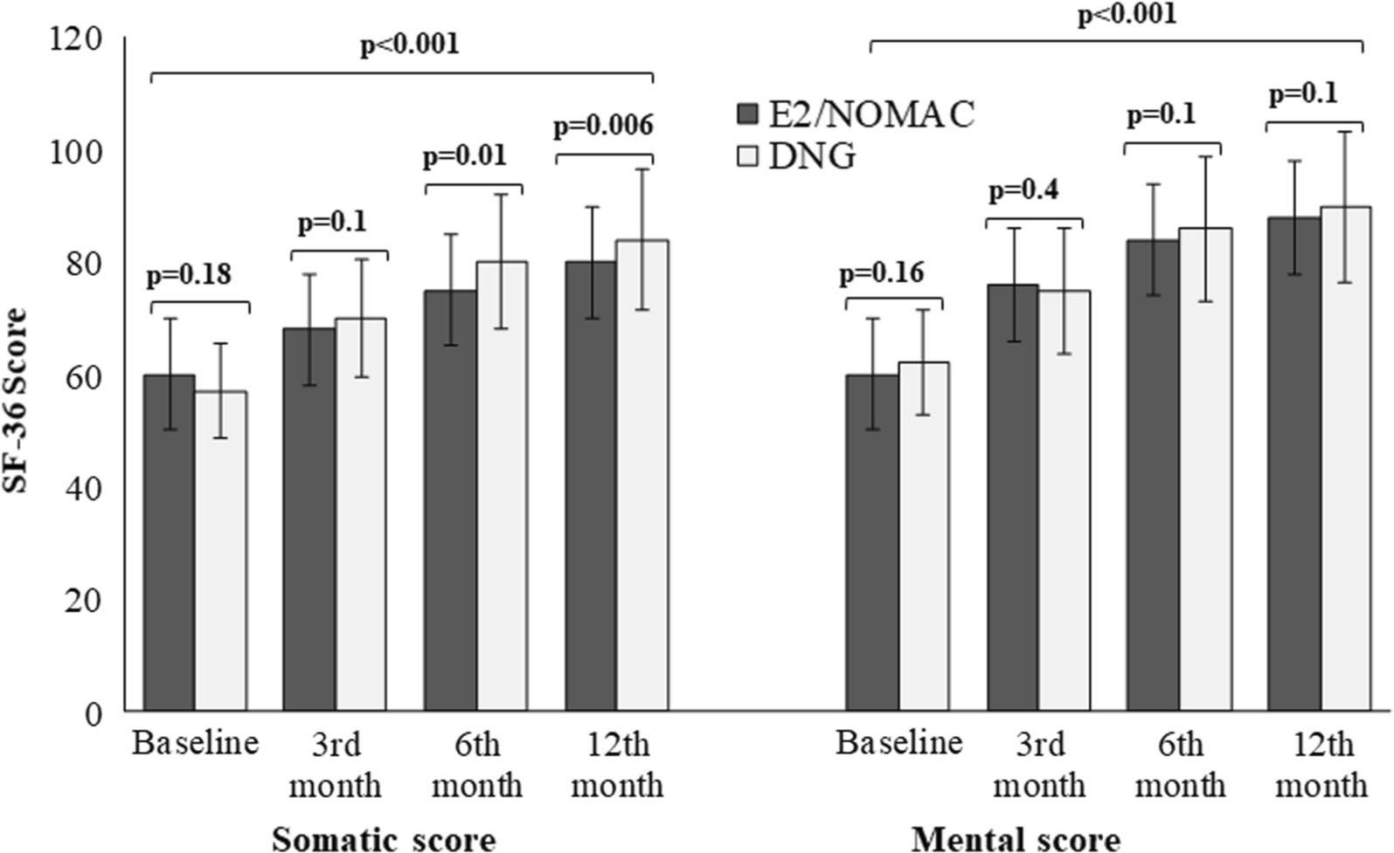
Quality of Life intragroup and intergroup comparison of women with endometriosis-associated pain symptoms at baseline and at 3, 6 and 12 months of 24/4 regimen 17β-estradiol (1.5 mg) and Nomegestrol Acetate (2.5 mg) (E2/NOMAC) combined oral contraceptive, or of Dienogest (DNG) 2 mg daily

The QoL of the women of both groups improved from the 3-month to the 12-month follow-ups (*p* < 0.001) (Fig. 3).

The intergroup differences were not statistically significant at each follow-up for mental scores (*p* ≥ 0.16; Cohen’s d ≤ 0.23), and at baseline and at the 3-month follow-up for the somatic score (*p* ≥ 0.18; Cohen’s d ≤ 0.23). However, women on DNG had a better somatic score than women on E2/NOMAC at both the 6-month (*p* = 0.01; Cohen’s d = 0.34) and 12-month follow-ups (*p* < 0.006; Cohen’s d = 0.39), even if the effect size between the groups was not statistically significant (Table 3).

**Table 3:** Intergroup comparison of SF-36 somatic and mental scores at baseline and at 3, 6 and 12 months of women on 24/4 regimen 17β-estradiol (1.5 mg) and Nomegestrol Acetate (2.5 mg) (E2/NOMAC) combined oral contraceptive, or on Dienogest (DNG) 2 mg daily

SF-36 somatic score	Baseline	3rd month follow-up	6th month follow-up	12th month follow-up
**A**				
E2/NOMAC Group	60 ± 10	68 ± 10	75 ± 8	80 ± 11
DNG Group	57 ± 18	70 ± 7	80 ± 19	84 ± 9
*p*	0.18 95% CI − 1.08 to 7.08	0.1 95% CI − 4.42 to − 0.42	0.01 95% CI − 9.08 to − 0.91	0.006 95% CI − 6.82 to − 1.17

SF-36 mental score	Baseline	3rd month follow-up	6th month follow-up	12th month follow-up
**B**				
E2/NOMAC Group	60 ± 7	76 ± 8	84 ± 8	88 ± 9
DNG Group	62 ± 12	75 ± 9	86 ± 9	90 ± 10
*p*	0.16 95% CI − 4.75 to 0.75	0.4 95% CI − 1.39 to 3.39	0.1 95% CI − 0.41 to 0.62	0.1 95% CI − 4.67 to − 0.67

The FSFI and FSDS improved from the 3- to the 12-month follow-ups in both groups, above the cut-off (*p* < 0.001). The FSFI intergroup differences were not statistically significant at the 3-month follow-up (*p* = 0.15; Cohen’s d = 0.20), but women on DNG had a better FSFI score than the women on E2/NOMAC at the 6-month (*p* = 0.005; Cohen’s d = 0.40) and 12-month follow-ups (*p* = 0.006; Cohen’s d = 0.39) (Table 4A).

**Table 4:** (A) Female Sexual Function Index (FSFI) and (B) Female Sexual Distress Scale (FSDS) intragroup and intergroup comparison of women with endometriosis-associated pain symptoms at baseline and at 3, 6 and 12 months of 24/4 regimen 17β-estradiol (1.5 mg) and Nomegestrol Acetate (2.5 mg) (E2/NOMAC) combined oral contraceptive, or of Dienogest (DNG) 2 mg daily

FSFI score	Baseline	3rd month follow-up	6th month follow-up	12th month follow-up	*p*
**A**					
E2/NOMAC Group	20.8 ± 1.9	27.5 ± 1.6	28.6 ± 2.5	30.2 ± 2.8	< 0.001
DNG Group	21.1 ± 1.2	27.8 ± 1.3	29.7 ± 2.9	31.3 ± 2.7	< 0.001
*p*	0.18 95% CI − 0.7 to 0.1	0.15 95% CI − 0.7 to 0.1	0.005 95% CI − 1.8 to − 0.3	0.006 95% CI − 1.8 to − 0.3	

FSDS score	Baseline	3rd month follow-up	6th month follow-up	12th-month follow-up	*p*
**B**					
E2/NOMAC Group	18.5 ± 1.5	13.6 ± 1.7	10.2 ± 1.8	10 ± 1.4	< 0.001
DNG Group	18.4 ± 1.3	11.3 ± 1.4	10.1 ± 1.6	9.8 ± 1.5	< 0.001
*p*	0.6 95% CI − 0.3 to 0.5	< 0.001 95% CI 1.8 to 2	0.68 95% CI − 0.4 to 0.6	0.33 95% CI − 0.2 to 0–6	

On the other hand, the FSDS score of the women on DNG was better than that of the women on E2/NOMAC only at the 3-month follow-up (*p* < 0.001; Cohen’s d = 1.4). In fact, at the 6-month (*p* = 0.68; Cohen’s d = 0.05) and 12-month (*p* = 0.33; Cohen’s d = 0.30) follow-ups there were no statistically significant intergroup differences (Table 4B). However, even with respect to FSFI and FSDS scores, the effect size between groups was not statistically significant.

Moreover, the FSFI scores showed a negative correlation with VAS values, more statistically significant in the E2/NOMAC group (r − 0.99; *p* < 0.004) than in the DNG group (r − 0.96; *p* < 0.03).

Finally, the intergroup satisfaction rate was similar at each follow-up. No woman of each group reported to be dissatisfied or very dissatisfied during the treatment.

## Discussion

The primary endpoint of this randomized study was to investigate the efficacy of E2/NOMAC or DNG intake in women with endometriosis-associated CPP, during 12 months of treatment. Firstly, women of both groups experienced a gradual and meaningful improvement in pain syndrome, namely CPP, dysmenorrhea, and dyspareunia, throughout the study. This study found an improvement in all physical and mental aspects of the QoL. The Endometriosis Health Profile-30 (EHP-30) questionnaire is more specific and should be adopted in cases where the diagnosis of endometriosis is ultrasound or surgically confirmed [16]. The SF-36 questionnaire was chosen to use in the current investigation because the study was based on the pain symptoms suggestive of endometriosis diagnosis. A concomitant and gradual improvement of sexual function and a reduction of sexual distress were observed in both groups. The progressive reduction of the pain syndrome reported by women over the treatment period could have contributed to further improving their QoL and their sexual life. Although the women of both groups had an improvement in their endometriosis-associated pain, from the 3-month follow-up to the end of the study, women on DNG had a better improvement in their pain symptoms at the 6-month follow-up than those on E2/NOMAC. Moreover, the intergroup comparison showed a better improvement of QoL and sexual function in women who were using DNG than in those on E2/NOMAC, mainly for the somatic scores at the 6- and 12-month follow-ups. Although the strength of statistical significance of the effect size was medium to large when calculated by Cohen's *d,* the efficacy of both treatments was shown to be high towards all endpoints. This could depend on the different personal/subjective processing of the improvement perception by the women.

A major reason for excluding women from enrollment in the current study was evidence of endometrioma or infiltrative lesions. In fact, the speculative objective of the study was to recognize, on the basis of symptoms and according to the ESHRE guidelines, women with possible endometriosis, and to treat them even before submitting them to invasive diagnosis, or even worse, neglecting them from a medical point of view. The effects of DNG on ultrasonographical diagnosed endometrioma are well known. Recently, some authors have reported a reduction of endometrioma volume and pain symptoms of women on DNG [17].

A conservative approach is usually shared by a multidisciplinary team. Surgical treatment will be chosen only when hormonal treatments are not effective or contraindicated, or the lesion subverts the bladder or bowel anatomy and function, or impairs fertility [18].

The design of the study was based on critical observation of the survey length. In fact, efficacy studies are usually limited to 3–6 months. By adopting such a design, we often cannot know what longer-term effects and benefits the treatment may have and how many people discontinue it [19].

Today, several progestogens, although not approved to treat pain syndrome associated with endometriosis, are widely prescribed [3]. DNG, unlike other progestogens, has been approved for medical treatment of endometriosis [9], by continuous or extended regimens [20]. DNG 2 mg daily has been shown to have insufficient contraceptive activity [21], therefore women who do not wish to become pregnant could use an estrogen-progestogen contraceptive. One of its catabolic characteristics consists in its half-life of 10 h, in a continuous or extended regimen; therefore, it has to be administered on a continuous or extended regimen, associated or combined with an estrogen. Treatment of endometriosis-associated pain symptoms should exclude hormone-free interval regimens. In fact, if this were the regimen, women may complain of symptoms returning during the hormone-free interval. Some authors showed that DNG combined with estradiol valerate in a 26/2 four-phase association was effective in reducing the pain syndrome [22]. The proliferative activity of the endometrial epithelium induced by estrogen is well known, as well as the inhibitory activity of progesterone towards its proliferation. The synchronous activity of the two steroids in ectopic endometrial tissue is not respected. In fact, the unbalanced activity between the two steroids, with estrogen predominance, promotes a chronic inflammatory state. The inability of progesterone to balance the proliferative activity of estrogen depends on an altered or reduced expression of its receptor in endometriosis tissues [23]. Reduced progesterone activity is accompanied by low levels of 17β-hydroxysteroid dehydrogenase (17β-HSD) type 2 production, whereby E2 is not converted to estrone, its biologically less potent metabolite; moreover, in endometriosis tissue there is an high production of p450 aromatase that increases the E2 level [24]. Increased neuroinflammation and neoangiogenesis due to increased E2, support the persistence of CPP [25]. Because of what has been mentioned above, when E2 is used in hormonal contraception, it could have metabolic and tissue effects that are less than those of EE. Indeed, it is interesting to know that 5 mg of EE is equivalent to about 1 mg of E2. Therefore, the common usage of low-dose OCs containing 20 to 30 mg of EE is equivalent to 4 to 6 times the physiologic dose of E2 [26].

The main therapeutic activity of progestogens in endometriosis is due to progesterone receptor signaling, which induces the downregulation of estrogen receptors [27]. DNG is able to inhibit aromatase expression and, as a result, local estrogen production is reduced [28]. Moreover, DNG inhibits the expression of 17β-HSD type 1, the enzyme that catalyzes the reduction of estrone to estradiol, and upregulates the expression of the oxidative 17β-HSD type 2, which inactivates estradiol [29].

NOMAC is a 19-norprogesterone derivative that binds specifically to the progesterone receptor; it has strong antiestrogenic effects, inhibiting 17β-HSD type 1, with a consequent reduction of conversion of estrone to estradiol [30]. Moreover, it reaches a peak serum concentration within 4 h after oral administration and, unlike DNG, its half-life is approximately 50 h [8]. Thus, in the 24/4 E2/NOMAC OC regimen, the steroidal activity of NOMAC is able to cover the 4-day hormone-free interval. In fact, women on E2/NOMAC usually have reduced bleeding, and some women experience amenorrhea [31]. In our study, 30.3% of women had amenorrhea, however, less than the women on DNG (65.3%). Reduction in bleeding, or even more so, amenorrhea observed in both groups could decrease or disrupt, respectively, the retrograde flow of menstrual tissue through the fallopian tubes, which is one of the most established hypotheses of endometriosis pathogenesis [32]. However, in our study, 10.1% and 10.3% of women on E2/NOMAC and on DNG, respectively, discontinued treatment for irregular bleeding. Spotting is one of the main reasons for discontinuing hormone treatments, even when women have pain syndrome benefits [33]. The women who discontinued were placed in a treatment group with CHCs having other regimens, not considered in this study.

It is important to consider that CPP and dysmenorrhea usually start during adolescence, but treatments are often started several years later and are not always adequate to limit or reduce the progression of endometriosis. Early diagnosis and treatment are essential in order to decrease neoangiogenesis and neuroinflammation and thus the chronic inflammatory status, and preserve future fertility [34]. Pain symptoms usually reappear when estrogen-progestin or only progestin intake is discontinued. In fact, progestins and combined hormonal contraceptives do not eliminate endometriotic lesions but induce their quiescence [35]. Consequently, their usage has to be long-term. However, unlike progestin-only contraceptives, biological data support the results that prolonged use of estrogen-progestogen contraception could promote a progression of endometriotic lesions. Consequently, some authors support the use of progestin-only contraceptives rather than OCs, considering them as first-line therapy for treating endometriotic CPP [36].

In all the women using E2/NOMAC or DNG, QoL and sexual function improved. Even if receptor binding studies showed that DNG has approximately one-third of the anti-androgenic activity of cyproterone acetate, and could affect the libido of long-term users, while recent studies showed that the quality of sexual life, particularly, improves during DNG treatment of women with endometriosis [19]. On the other hand, women using E2/NOMAC had benefits on their sexual function and sexual distress [37]. In fact, E2 could have less antiandrogenic activity than EE by inducing less SHBG improvement [38]. In addition to the effects due to the biological activity of steroids, the reduction of painful symptoms could favor subjective well-being, such as improving the quality of sexual life. In fact, although DNG has antiandrogenic activity that could reduce sexual desire and arousal, the decrease in painful symptoms, especially dyspareunia, may be capable of promoting satisfactory sexual function [19]. CPP is associated with substantial personal and economic burdens and increased risk for behavioral and mental disorders. Therefore, it is essential not only to control the pain syndrome but also to take care in planning treatments that allow the woman to have a QoL and a sexual function adequate to her expectations. Beyond the areas we take into consideration, multidisciplinary treatment is desirable. The biopsychosocial model of pain, from diagnosis to treatment, is increasingly adopted, depending on the phenotypic characteristics and the therapeutic expectations of each subject [39]. Such a study design could be used in future investigations.

## Conclusion

Our study was based on the clinical diagnostic symptoms of CPP associated with endometriosis, and not on a laparoscopic diagnosis, which remains the gold standard. This could be a limit of our study. However, today, laparoscopy is a very limited investigation for the diagnosis of endometriosis, and hormone therapy is initiated based on the painful symptoms reported by the woman [40].

Nevertheless, laparoscopic diagnosis to confirm endometriosis could be taken into consideration in future full studies.

The design of our study did not consider the contraceptive needs of the women enrolled, as it was a randomized study. In fact, each woman was advised that DNG should not be considered a contraceptive. On the other hand, the women enrolled had no contraceptive needs. Moreover, we need to expand the number of investigations using E2/Progestogen rather than EE/Progestogen contraceptives to understand if the effectiveness of the former is better or equal to that of the latter.

## Data Availability

The datasets generated and analyzed during the current study are not publicly available to maintain the privacy of the participants, but are available from the corresponding author upon reasonable request and with permission of the Ethics Committee.
